# Cardiovascular Safety of Febuxostat and Allopurinol in Hyperuricemic Patients With or Without Gout: A Network Meta-Analysis

**DOI:** 10.3389/fmed.2021.698437

**Published:** 2021-06-15

**Authors:** Shengzhao Zhang, Ting Xu, Qingyang Shi, Sheyu Li, Ling Wang, Zhenmei An, Na Su

**Affiliations:** ^1^Department of Pharmacy, West China Hospital, Sichuan University, Chengdu, China; ^2^West China School of Pharmacy, Sichuan University, Chengdu, China; ^3^Department of Guideline and Rapid Recommendation, Cochrane China Center, MAGIC China Center, Chinese Evidence-Based Medicine Center, West China Hospital, Sichuan University, Chengdu, China; ^4^Department of Endocrinology and Metabolism, West China Hospital, Sichuan University, Chengdu, China

**Keywords:** febuxostat, allopurinol, hyperuricemia, network meta-analysis, cardiovascular safety, Bayesian framework

## Abstract

**Background:** Hyperuricemia is a common metabolic disease and has become a public health problem because of its increasing prevalence and association with comorbidities. Allopurinol and febuxostat are recommended as the first-line treatments for hyperuricemia and gout. But cardiovascular safety between febuxostat and allopurinol is still controversial. The purpose of this study is to compare the cardiovascular safety of XOIs and placebo in hyperuricemic patients with or without gout.

**Methods:** PubMed, Embase via OVID, Cochrane Library, CNKI, Wanfang, and VIP were searched from their earliest records to February 8th 2021. ClinicalTrials.gov was also searched for unpublished data. The reference lists of included studies and relevant review articles investigating the cardiovascular safety of XOIs in hyperuricemia patients are screened for potentially eligible studies. Randomized controlled trials (RCTs) evaluating allopurinol (100~900 mg/d), febuxostat (20~120 mg/d), or placebo for hyperuricemia were included. The outcomes were incidence of MACE, non-fatal MI, non-fatal stroke, and cardiovascular death. We conducted a Bayesian random-effects network meta-analysis on the included randomized controlled trials using the Markov Chain Monte Carlo simulation method. The grading of recommendations assessment, development, and evaluation (GRADE) approach was used to assesses the certainty of the evidence.

**Results:** Ten RCTs with 18,004 participants were included. The network estimates showed that there was no significant difference observed among febuxostat, allopurinol, and placebo regarding outcomes. The certainty of the evidence ranged from very low to moderate. The probabilities of rankings and SUCRA showed that compared to placebo, febuxostat, and allopurinol might prevent adverse cardiovascular events.

**Conclusion:** Febuxostat is not associated with increasing risk of adverse cardiovascular events compared to allopurinol; and compared to placebo, whether febuxostat and allopurinol reduce the risk of adverse cardiovascular events remains uncertain.

## Introduction

Hyperuricemia is a metabolic disease caused by disorders in purine metabolism or reduced uric acid excretion and develops into gout with prolonged elevation of serum urate (1). Hyperuricemia is “traditional risk factor” for cardiovascular diseases with hyperlipidemia, hypertension, and diabetes (2–5). However, the causation between hyperuricemia and cardiovascular diseases remains debated (6). Although this causation was suggested by a recent Mendenlian randomization study (7), cohort studies showed a U-shaped association between sUA (serum uric acid) and the incidence of cardiovascular diseases (8). This means that both elevated and very low levels of sUA can be linked to cardiovascular risk.

To prevent gout flares and other comorbidities, urate-lowering drugs are commonly used for patients with hyperuricemia (9). There are three main categories of urate-lowering drugs: xanthine oxidase inhibitors (XOIs) (e.g., allopurinol and febuxostat), uricosurics (e.g., probenecid, benzbromarone, and lesinurad), and recombinant uricase (e.g., pegloticase). In many countries, allopurinol and febuxostat are recommended as the first-line treatments for hyperuricemia and gout (10–12). Febuxostat is often considered more effective in urate-lowering than allopurinol, and febuxostat used for patients with renal dysfunction (30 ml/min < GFR < 89 ml/min) does not require dose adjustment (13–15). However, it remains unclear whether urate-lowering drugs may improve long-term cardiovascular outcomes. A recent trial linked long-term febuxostat to a mildly increased risk of cardiovascular death (16), when the results were not validated in another large trial (17). In this systematic review, we focus on the cardiovascular outcomes of XOIs (e.g., allopurinol and febuxostat) for hyperuricemic patients using a network meta-analysis.

## Methods

We conducted this study in accordance with the guidelines of the Preferred Reporting Items for Systematic Reviews and Meta-analyses (PRISMA) checklist for network meta-analyses (18). This network meta-analysis was registered on International Prospective Register of Systematic Review (PROSPERO, CRD42021244788).

### Literature Search and Eligible Criteria

We comprehensively searched the PubMed, Embase via OVID, Cochrane Library, CNKI (China National Knowledge Infrastructure), Wanfang, and VIP electronic databases to identify relevant studies published until February 8th, 2020. ClinicalTrials.gov was also searched for unpublished data. The reference lists of included studies and relevant review articles investigating the cardiovascular safety of XOIs in hyperuricemia patients are screened for potentially eligible studies. Based on the PICOS (Participants, Intervention, Comparison, Outcome, and Study design) framework, the key terms searched in this study were hyperuricemia, drug therapy, febuxostat, allopurinol, and randomized controlled trial.

The inclusion criteria were as follows: (a) Participants: adult patients (>18 years) with a diagnosis of hyperuricemia with or without gout. (b) Interventions/comparisons: febuxostat, allopurinol or placebo. (c) Outcomes: major adverse cardiovascular events (MACE; composite endpoint of nonfatal myocardial infarction, nonfatal stroke, and cardiovascular death), nonfatal myocardial infarction (MI), nonfatal stroke and cardiovascular death. (d) Study design: randomized controlled trials of 4 weeks or more of treatment and follow-up duration. The exclusion criteria were as follows: (a) acute gout or secondary gout, (b) animal experiments, (c) poor-quality studies (random sequence generation, allocation concealment, and blinding approaches are all assessed as high risk based on the Cochrane bias risk tool), (d) patients with moderate or severe hepatic impairment (value, ascites, lower limb edema, icterus, alanine aminotransferase (ALT) or aspartate aminotransferase (AST) >3× reference or increased prothrombin time >2× reference value), severe renal impairment (eGFR <15 mL/min), or advanced cancer, and (e) studies published in a language other than Chinese or English.

### Screening Process, Data Extraction, and Risk of Bias

Two authors (SZ, TX) independently screened titles and abstracts based on the inclusion and exclusion criteria identified in section Literature Search and Eligible Criteria. Studies identified as potentially relevant were then checked via full-text review. This screening process continued until all remaining literature was checked. Discrepancies were resolved by discussion between these two researchers and, if necessary, by consulting the third member (NS) of our team.

Two reviewers (SZ, TX) independently extracted individual study data and entered them into an electronic database. Discrepancies were resolved through discussion with a third reviewer (NS). These study data included the first author's name, publication year, interventions/comparisons, outcomes, durations, and baseline characteristics of patients including sex and age. We used Intention-to-treat sample sizes when available.

Two members (SZ, TX) of the research team used the Cochrane bias risk tool by RevMan version 5.4 to independently assess the risk of bias of all included studies (19). Any discrepancies were resolved by discussion with a third reviewer (NS).

### Treatment Nodes

Treatment nodes were grouped by drugs. Only the common dose of each drug was eligible. Considering the different recommended doses of drugs in different countries, the common dose was defined as 20–120 mg/d for febuxostat, and 100–900 mg/d for allopurinol. We drew network plots with the *multinma* package in R (version 4.0.3) (20).

### Statistical Analysis

We conducted a network meta-analysis of randomized controlled trials that assessed the cardiovascular safety of febuxostat and allopurinol using a random-effects model and consistency model. This analysis was estimated in a Bayesian framework (21). Odds ratios (ORs) and 95% credible intervals (CIs) were used to report the effect size for assessing cardiovascular safety. We used the Markov chain Monte Carlo method (22), built up four chains, and set 160,000 iterations after an initial burn-in of 40,000 and a thinning of one. We assessed local incoherence and obtained indirect estimates by node splitting models (23). We calculated the probabilities of the surface under the cumulative ranking curve (SUCRA) to rank treatments (24). We performed sensitivity analysis by excluding trials without double blinding.

In this analysis, *P* < 0.05 was considered statistically significant. All statistical analyses were conducted using the *gemtc* package in R (Version 4.0.3) (25).

### The Certainty of Evidence

The certainty of evidence was assessed by the Grading of Recommendations Assessment, Development, and Evaluation (GRADE) approach for network meta-analysis (26–28). Two members of the research team assessed the certainty for each comparison as high, moderate, low, or very low, based on consideration of the risk of bias, incoherence, inconsistency, indirectness, intransitivity, publication bias, and imprecision. Discrepancies were resolved by discussions.

## Results

### Characteristics of Eligible Studies

**Figure 1:**
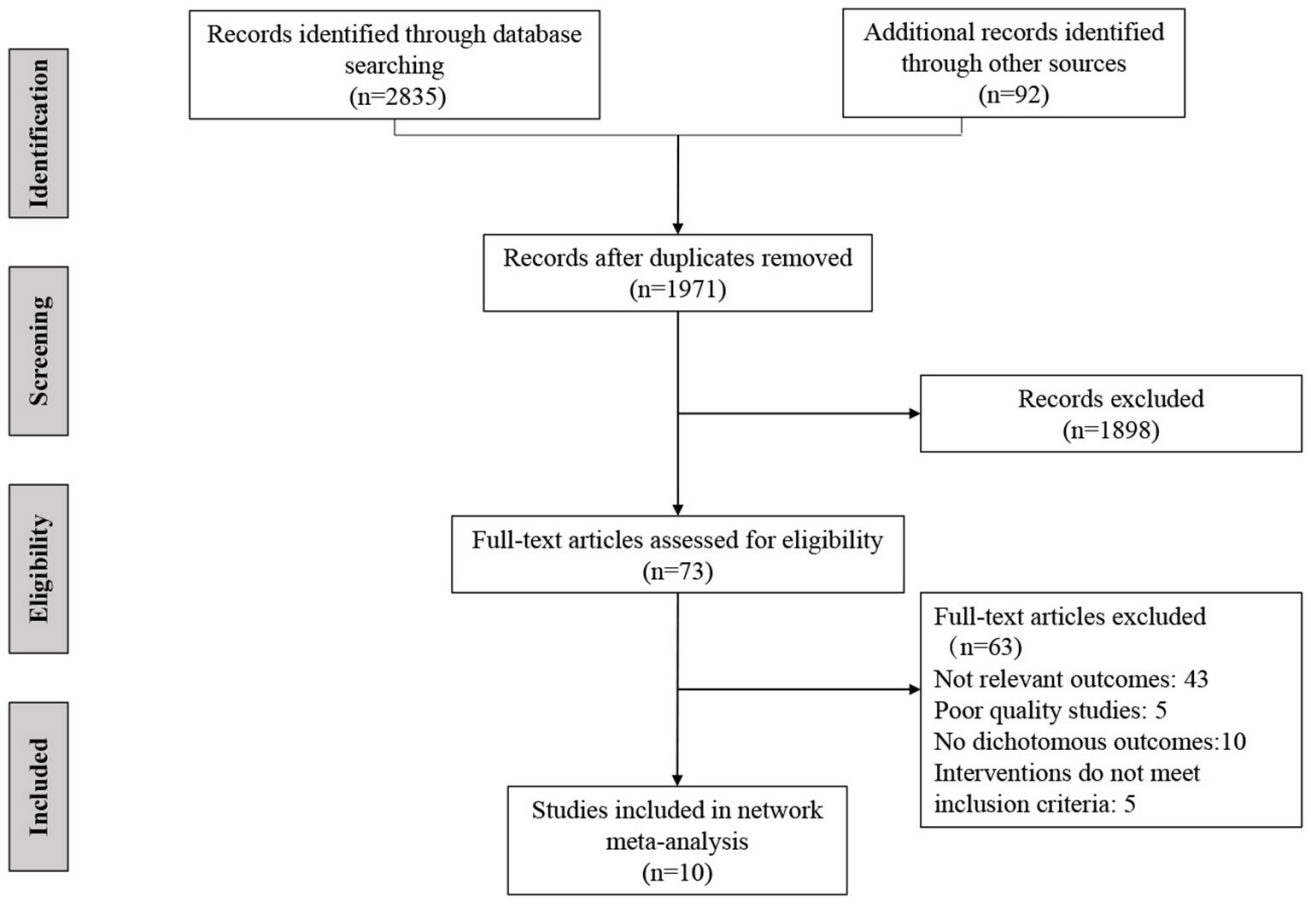
Flow diagram of literature search and selection.

After screening 1,971 citations and 73 full texts, 10 randomized controlled trials met the inclusion criteria in our systematic review (Figure 1). The included trials were conducted in 4 countries or regions (USA, Canada, Japan, and Europe), and most of trials were registered (9/10, 90%), all of which were published in English. Among the included studies, seven were two-arm studies and three were three-arm studies. Table 1 presents the baseline characteristics of the included studies (16, 17, 29–36). The mean age of the participants was ranged from 50 to 76 years old, and the proportion of males ranged from 69% to 97%. Six trials assessed all four outcomes, two trials assessed three outcomes, and two trials assessed two outcomes. The length of follow-up ranged from 24 to 312 weeks.

**Table 1:** Characteristics baseline of randomized controlled trials.

**Study ID**	**Register number/trial name**	**Location**	**No. of patients**	***N***	**Intervention**	**Age**	**Male (%)**	**Outcomes**	**Length of follow-up (weeks)**
White et al. (16)	NCT01101035	USA	7,190	3,098	Febuxostat 40~80 mg/day	64.3 ± 9.6	84.1%	①②③④	312
				3,092	Allopurinol 300~600 mg/day	64.7 ± 9.6	83.8%		
Mackenzie et al. (17)	EudraCT 2011-001883-23	Europe	6,128	3,063	Febuxostat 80~120 mg/day	71.0 ± 6.4	85.5%	①②③④	312
				3,065	Allopurinol 100~900 mg/day	70.9 ± 6.5	85.0%		
Tanaka et al. (29)	UMIN0000112911	Japan	483	239	Febuxostat 10~40 mg/day	69.1 ± 10.1	79.5%	①②③④	104
				244	Placebo	69.1 ± 10.7	81.1%		
Becker et al. (30)	FACT	USA, Canada	760	256	Febuxostat 80 mg/day	51.8 ± 11.7	95.0%	①④	52
				251	Febuxostat 120 mg/day	52.0 ± 12.1	97.0%		
				253	Allopurinol 300 mg/day	51.6 ± 12.6	96.0%		
Kojima et al. (31)	NCT01984749	Japan	1,070	537	Febuxostat 10~40 mg/day	75.4 ± 6.7	69.1%	①②③④	156
				533	Allopurinol 100~900 mg/day	76.0 ± 6.5	69.0%		
Kimura et al. (32)	UMIN000008343	Japan	441	219	Febuxostat 10~40 mg/day	65.3 ± 11.8	77.6%	①②③	108
				222	Placebo	65.4 ± 12.3	77.0%		
Dalbeth et al. (33)	NCT010783	USA	314	157	Febuxostat 40 mg/day	51.4 ± 12.4	91.1%	①②④	104
				157	Placebo	50.1 ± 11.7	92.4%		
Saag et al. (34)	NCT01082640	USA	96	32	Febuxostat 30 mg BID	67.3 ± 11.11	78.1%	①②③④	52
				32	Febuxostat 40~80 mg QD	63.6 ± 8.15	81.3%		
				32	Placebo	66.3 ± 12.05	81.3%		
Givertz et al. (35)	NCT00987415	USA	253	128	Allopurinol 100~600 mg/day	63.7 ± 15.0	86.0%	①④	24
				125	Placebo	62 ± 14.25	78.0%		
Becker et al. (36)	NCT00430248	USA	2269	757	Febuxostat 40 mg/day	52.5 ± 11.68	95.4%	①②③④	26
				756	Febuxostat 80 mg/day	53.0 ± 11.79	93.9%		
				756	Allopurinol 200~300 mg/day	52.9 ± 11.73	93.8%		

*①, MACE (composite endpoints of non-fatal MI, no-fatal stroke or cardiovascular death); ②, non-fatal MI (myocardial infarction); ③, non-fatal stroke; ④, cardiovascular death*.

### Risk of Bias of Included Studies

The assessment of the risk of bias of the included studies is presented in Supplementary Figure 2. Three studies had high risk in the domain of blinding of participants and outcome assessment (16, 29, 31). All other included studies were evaluated at low risk of bias in all domains.

### Results of Network Meta-analysis

**Figure 2:**
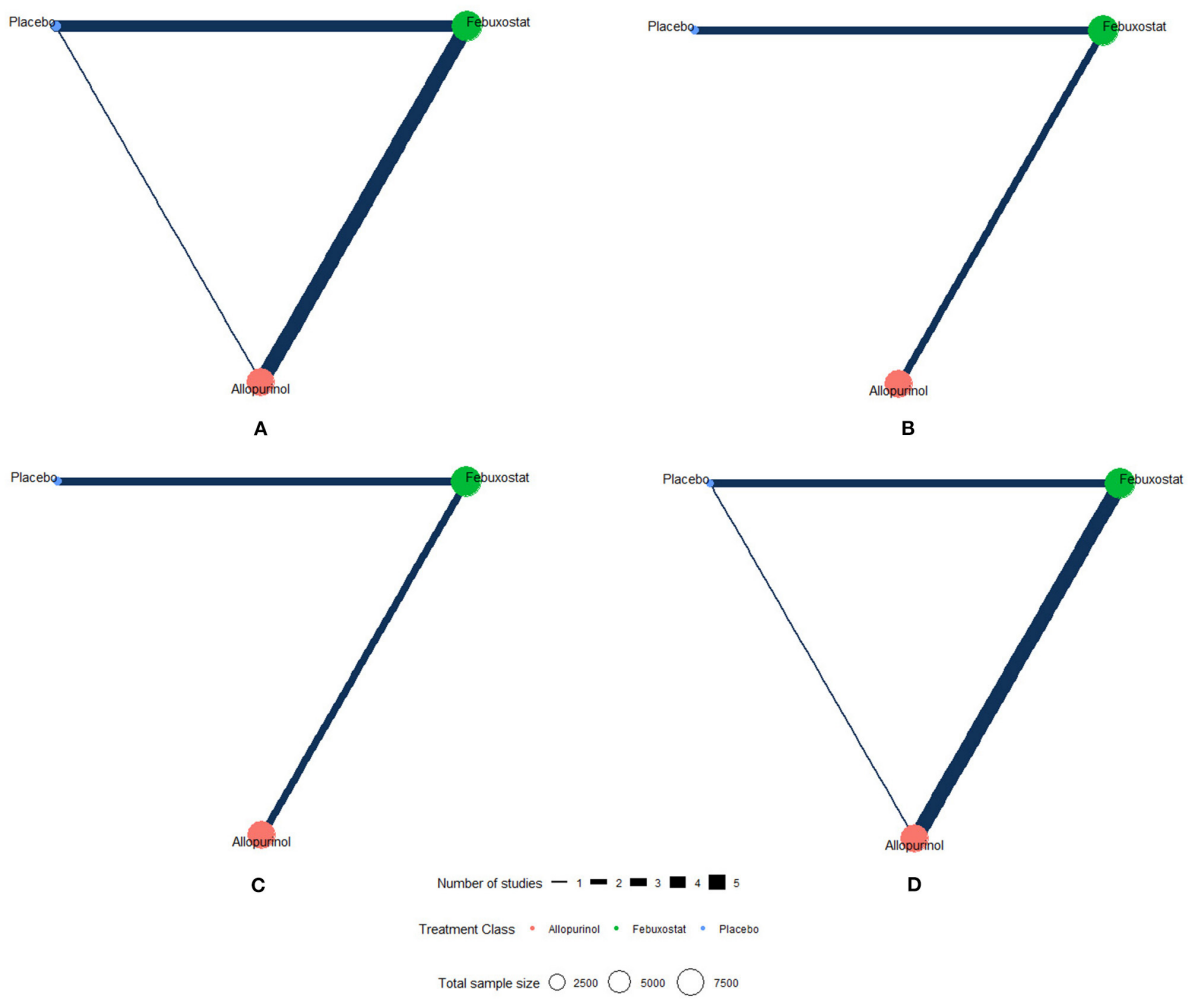
Network plots of each outcome. The network of eligible studies for different outcomes. Different color of nodes represents different treatment. The node sizes correspond to the number of participants that investigated the treatments. The thickness of edges represents the number of trials. A lack of line indicates that there were no head-to-head trials between two treatments for this outcome. **(A)** MACE (major adverse cardiovascular events), **(B)** non-fatal MI (myocardial infarction), **(C)** non-fatal stroke, and **(D)** cardiovascular death.

**Figure 3:**
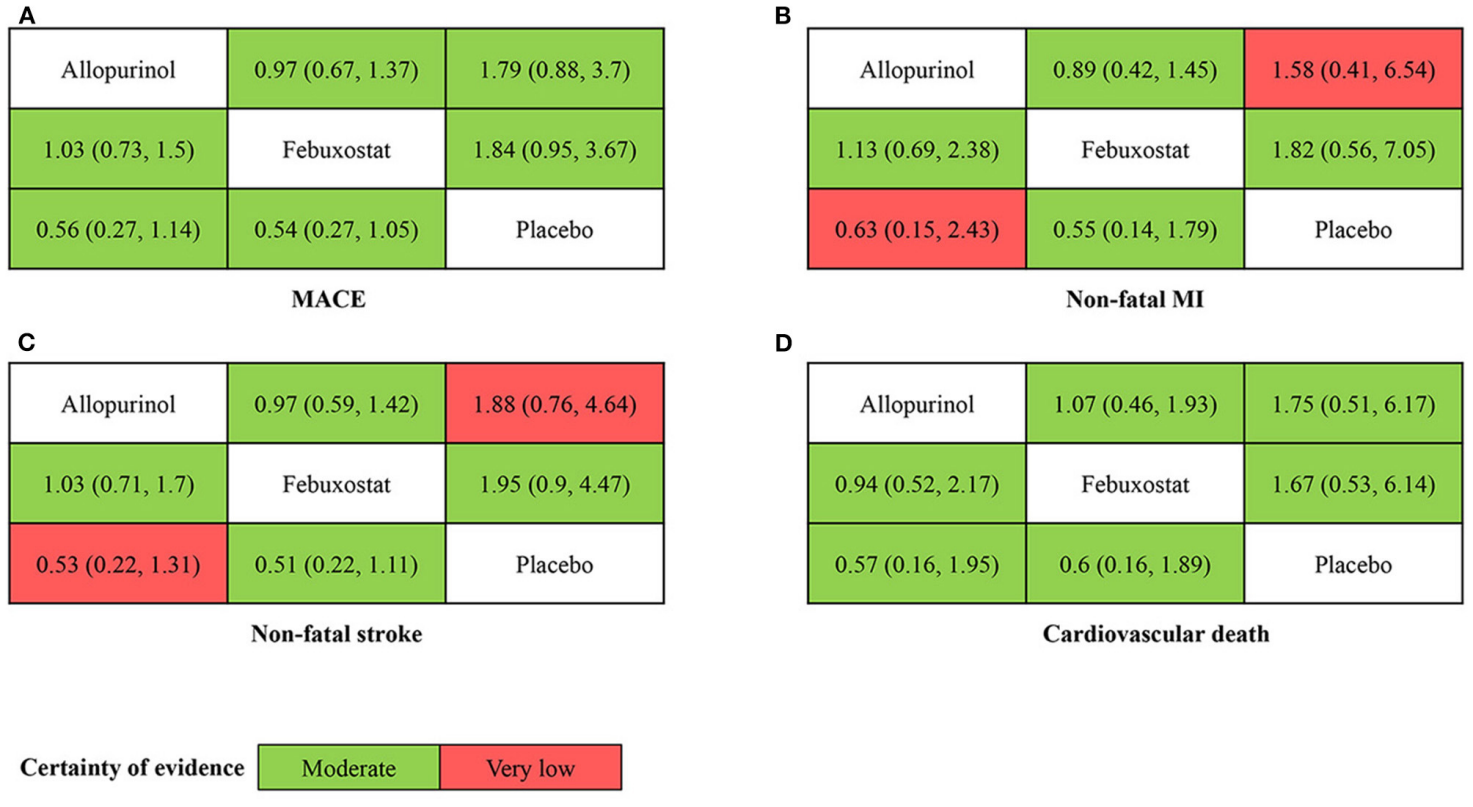
League table of each outcome. Each number is an odds ratio (=column/row), and 95% confidence interval. Different color represents different certainty of evidence. OR < 1 favors the drug in the column. **(A)** MACE (major adverse cardiovascular events), **(B)** non-fatal MI (myocardial infarction), **(C)** non-fatal stroke, and **(D)** cardiovascular death.

The network plots of each outcome are presented in Figure 2. Figure 3 presents the results of our network meta-analysis and the certainty of evidence for all network estimates. Detailed results of the network meta-analysis and the certainty of evidence for all comparisons and outcomes are presented in the Supplementary Table 3. Detailed results of node split analysis are provided in the Supplementary Figure 3. The results of the sensitivity analysis are presented in the Supplementary Table 5.

#### MACE

Ten randomized controlled trials including 18,004 subjects reported the incidence of MACE. The intervention nodes included in this network meta-analysis were allopurinol, febuxostat, and placebo. There were no significant differences in either pairwise or network estimates. The certainty of evidence was moderate for all comparisons.

The global *I*^2^ of pairwise was 10.7% and the global *I*^2^ of consistency model was 0%. The node split analysis showed that the results were consistent. The results of sensitivity analysis were mostly similar to the results of main analysis.

#### Non-fatal MI

Eight randomized controlled trials including 16 991 subjects reported the incidence of non-fatal MI. The intervention nodes included were allopurinol, febuxostat, and placebo. There were no significant differences in either pairwise or network estimates. The certainty of the evidence was moderate for febuxostat compared with allopurinol, placebo compared with febuxostat, and very low for placebo compared with allopurinol.

The global *I*^2^ of pairwise was 0% and the global *I*^2^ of consistency model was 0%. There was no node split analysis of this outcome due to no loop. The results of sensitivity analysis were similar to the results of main analysis.

#### Non-fatal Stroke

Seven randomized controlled trials including 16 677 subjects reported incidence of non-fatal stroke. The intervention nodes included were allopurinol, febuxostat and placebo. There were no significant differences in either pairwise or network estimates. The certainty of the evidence was moderate for febuxostat compared with allopurinol, placebo compared with febuxostat, and very low for placebo compared with allopurinol.

The global *I*^2^ of pairwise was 3.9% and the global *I*^2^ of consistency model was 3.9%. There was no node split analysis of this outcome due to no loop. The results of sensitivity analysis were similar to the results of main analysis.

#### Cardiovascular Death

Nine randomized controlled trials including 17,563 subjects reported incidence of cardiovascular death. The intervention nodes included were allopurinol, febuxostat, and placebo. There were no significant differences in either pairwise or network estimates. The certainty of the evidence was moderate for all comparisons.

The global *I*^2^ of pairwise was 23.6% and the global *I*^2^ of consistency model was 13.7%. There was no node split analysis of this outcome due to no loop. The results of sensitivity analysis were similar to the results of main analysis.

### Rankings and SUCRA

**Figure 4:**
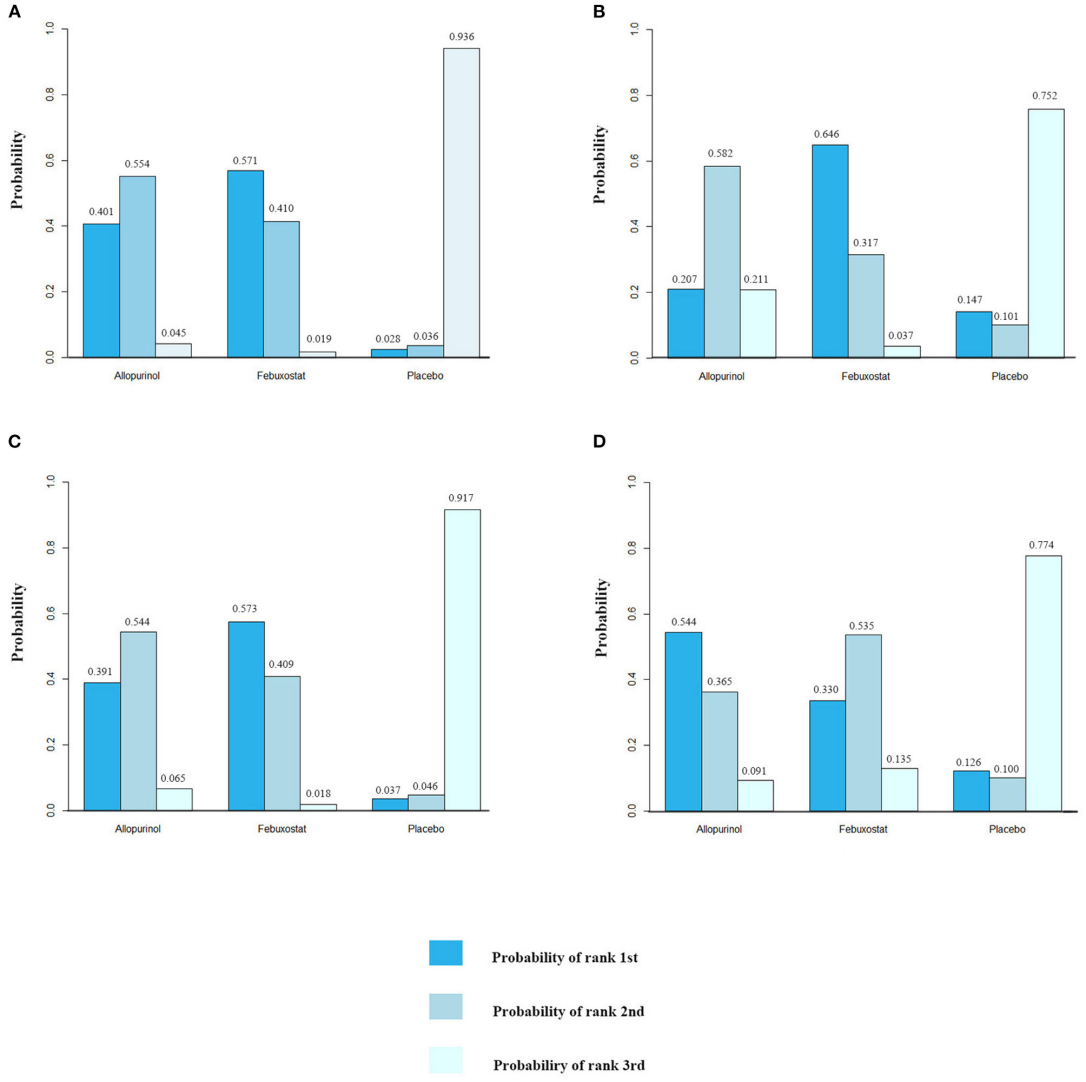
Rank diagram of febuxostat, allopurinol, and placebo for different outcomes. The numbers on the vertical axis represent the probability. **(A)** MACE (major adverse cardiovascular events), **(B)** non-fatal MI (myocardial infarction), **(C)** non-fatal stroke, and **(D)** cardiovascular death.

**Figure 5:**
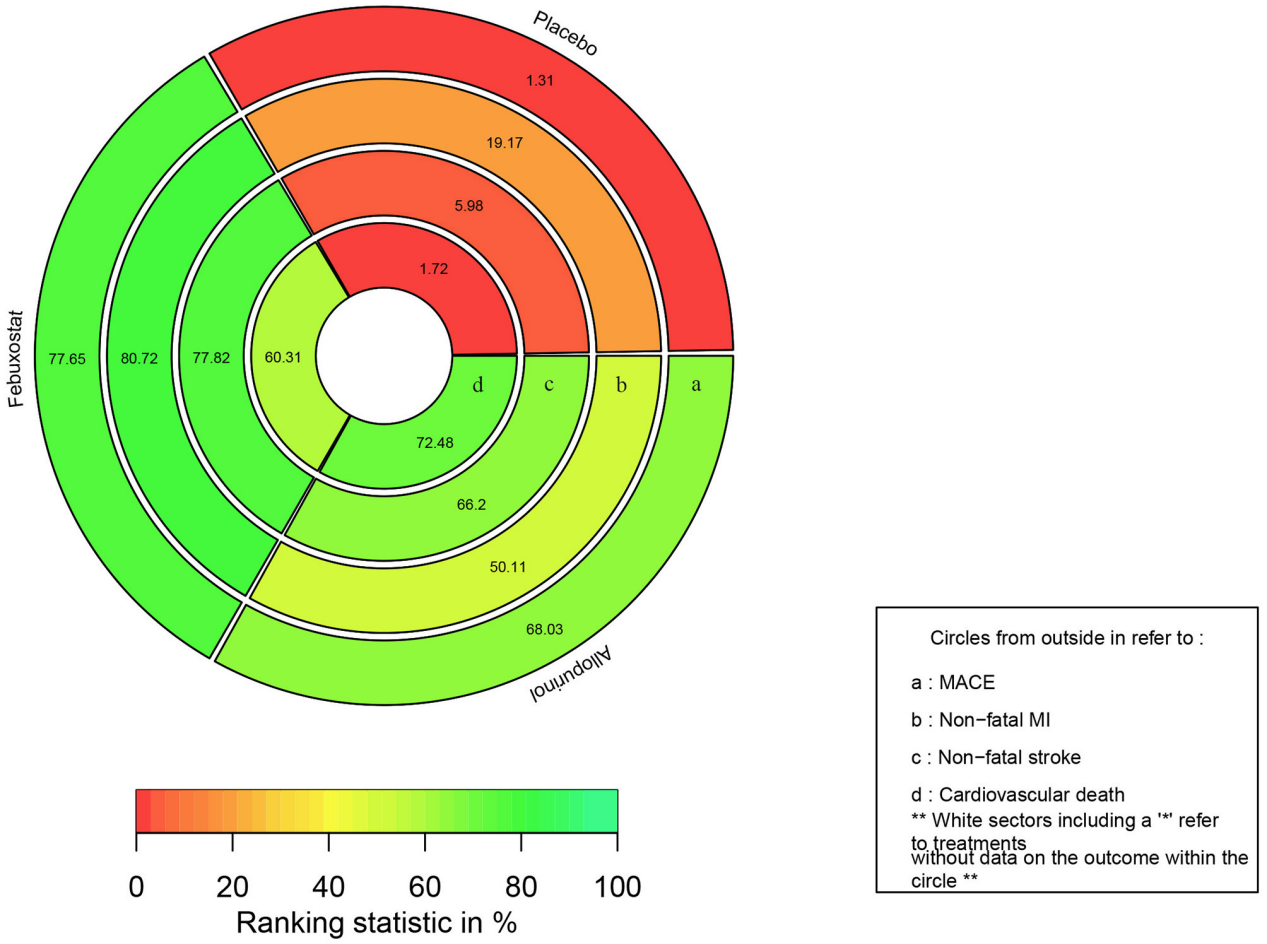
Rank-heat plot based on SUCRA. Each sector is colored according to the SUCRA (Surface under the cumulative ranking) value of the corresponding treatment and outcome. The scale consists of the transformation of three colors: red (0%), yellow (50%), and green (100%), and each color is associated with a different evaluating indicator. MACE, major adverse cardiovascular events; MI, myocardial infarction.

The rank probabilities of febuxostat, allopurinol, and placebo is shown in Figure 4, rank-heat plot based on SUCRA is presented in Figure 5. Detailed data is shown in Supplementary Table 4. According to Figures 4, 5, The differences of rank probabilities and SUCRA values between febuxostat and allopurinol are not significant; although network estimates showed no significant differences, the rank probabilities and SUCRA values of febuxostat and allopurinol display marked difference over placebo.

## Discussion

This network meta-analysis provides an overview of the evidence regarding the cardiovascular safety of febuxostat and allopurinol in people living with hyperuricemia. This result indicated that neither allopurinol nor febuxostat needs a concern of cardiovascular safety with very low to moderate certainty. Additionally, neither drug improves cardiovascular outcomes in people with hyperuricemia.

After FDA issued a boxed warning on febuxostat (37), more and more clinical trials have been devoted to focus on the cardiovascular safety of XOIs (17, 38–42). However, the conclusions of these trials were not unanimous. Until now, therefore, it remains unclear whether XOIs increase the risk of adverse cardiovascular events. The results of our network meta-analysis can provide reference for clinicians to treat patients with hyperuricemia using XOIs in terms of cardiovascular safety. To the best of our knowledge, this is the first Bayesian network meta-analysis of febuxostat, allopurinol, and placebo investigating cardiovascular outcomes as the primary outcomes. Before our studies, several systematic reviews were conducted to evaluate urate-lowering drugs in terms of cardiovascular safety. Two systematic reviews and meta-analyses that focused only on composite endpoints found that XOIs did not significantly reduce the risk of MACE (43, 44). This conclusion is consistent with our network estimates. Another systematic review and meta-analysis revealed that urate-lowering treatments might increase cardiovascular mortality (45), which included nonrandomized and retrospective studies, and that may be the reason why the conclusion of this study is different from ours. Although previous evidences suggested a significant benefit from allopurinol intake in increasing flow-mediated dilation in humans (46, 47), the results of our network meta-analysis did not indicate this cardiovascular benefit of allopurinol for patients with hyperuricemia. Actually, whether patients with asymptomatic hyperuricemia should be treated with urate lowering drugs remains controversial. Some guidelines recommended that urate lowering drugs should be used for asymptomatic hyperuricemia (9, 48), whereas others suggested that the benefits of urate lowering drugs would not overweigh the treatment costs or potential risks (14, 49). The results of our network meta-analysis indicated that for patients with hyperuricemia, XOIs did not increase the risk of adverse cardiovascular events.

The debates of CARES and FAST introduced heterogeneity in our study (16, 17). The reasons for the difference between the two results may be as follows: first, the baseline characteristics of the two trials were different, including the proportion of patients with established cardiovascular disease at baseline, the severity of cardiovascular disease, the severity of gout, and the proportion of patients with established urate lowering therapy. These differences at baseline might lead to different cardiovascular prognoses. Second, the doses of study medication were different. In CARES, doses of allopurinol were 200~600 mg/day, and doses of febuxostat were 40~80 mg/day. In FAST, doses of allopurinol were 100~900 mg/day, and doses of febuxostat were 80~120 mg/day. Although this difference reflected the different dose ranges for the two XOIs approved by regulatory agencies in North America and Europe, it is worth considering that the risk of adverse events generally increases with the increase of drug dose. Compared to FAST, lower doses of febuxostat in CARES lead to an increase in cardiovascular risk, in our opinion, therefore, the conclusion of FAST is more reliable. Third, the proportion of patients discontinued treatments, and the loss rate of follow-up of CARES was much higher than that of FAST, so the bias of CARES was greater than that of FAST, which further strengthens our view that the conclusion of FAST is more reliable. Fourth, differences in sponsors, practitioners and procedures may also lead to differences in the final conclusions.

The main limitation of our study is the limited quality of evidence. Limited quality of evidence is mainly due to imprecision which may be caused by the limited number of RCTs, resulting in the dependence on indirect comparisons of some network estimates. This problem would be resolved with the augmentation of high-quality RCTs. The second limitation is that our inclusion criteria were not highly strict so that some participants with comorbidities such as hypertension, diabetes, coronary artery diseases, and other diseases were included in our network meta-analysis. The third limitation is the short duration of some RCTs included in the present study. To obtain more reliable results, further studies may require longer duration. But our results are still convincing due to the following reasons. First, the dosage of the drug may be adjusted according to the actual situation. Second, patients with hyperuricemia often have different comorbidities. Third, node split analysis showed consistence in outcomes and heterogeneity is low. Fourth, the results of the sensitivity analysis were mostly similar to the results of our main analysis.

## Conclusion

This network meta-analysis suggests that neither allopurinol nor febuxostat increases or reduces the risk of major adverse cardiovascular events. However, current evidence does not support the cardiovascular benefits of XOIs. Due to the differences between large randomized controlled trials and real-world practice (50), real-world studies with long-term follow-up durations are warranted to validate the findings in our study.

## Data Availability Statement

The original contributions presented in the study are included in the article/Supplementary Material, further inquiries can be directed to the corresponding author/s.

## Author Contributions

SZ and TX were in charge of study design, data collection and interpretation, the quality assessment of evidence, and manuscript preparation. SL critically reviewed the manuscript and provided revisions. QS, LW, and ZA were involved statistical analysis. NS was involved in data collection, data interpretation, and the quality assessment of evidence. All authors contributed to the article and approved the submitted version.

## Conflict of Interest

The authors declare that the research was conducted in the absence of any commercial or financial relationships that could be construed as a potential conflict of interest.
