# Supplementary material for: Cardiovascular Safety of Febuxostat and Allopurinol in Hyperuricemic Patients With or Without Gout: A Network Meta-Analysis
Source: Front Med (Lausanne). 2021 Jun 15;8:698437. doi: 10.3389/fmed.2021.698437 (PMC8239361; doi:10.3389/fmed.2021.698437)
Supplement: Supplementary file 1 [file Table_1.docx]

**Table S1. Search strategy for English databases**

| Database | Search strategy |
| --- | --- |
| PubMed | ("Febuxostat"[MeSH Terms] OR "Allopurinol"[MeSH Terms]) OR (Febuxostat OR Allopurinol) AND "gout/drug therapy"[MeSH Terms]  Filters: Randomized Controlled Trial |
| Embase(via OVID) | ('febuxostat'/exp OR 'allopurinol'/exp) AND 'gout'/exp AND 'randomized controlled trial'/exp AND [randomized controlled trial]/lim |
| Cochrane Library | #1  [Gout] explode all trees and with qualifier(s): [drug therapy – DT]  #2 febuxostat OR allopurinol  #3 #1 AND #2 |
| ClinicalTrials | Condition or disease: Gout  Other terms: febuxostat OR allopurinol |
