# Supplementary material for: Cardiovascular Safety of Febuxostat and Allopurinol in Hyperuricemic Patients With or Without Gout: A Network Meta-Analysis
Source: Front Med (Lausanne). 2021 Jun 15;8:698437. doi: 10.3389/fmed.2021.698437 (PMC8239361; doi:10.3389/fmed.2021.698437)
Supplement: Supplementary file 2 [file Table_2.docx]

**Table S2. Search strategy for Chinese databases**

**中文数据库检索策略**

| Database | Search Strategy |
| --- | --- |
| Wanfang | 主题:(痛风) and 主题:(非布司他 OR 别嘌醇) |
| CNKI | [(主题=痛风) AND (主题=非布司他 + 别嘌醇)](https://kns.cnki.net/KNS8/AdvSearch?id=52&dbcode=SCDB&searchtype=gradeSearch&ishistory=1) AND (主题=对照试验 + 随机对照 + 系统评级 + Meta分析) |
| VIP | 题名或关键词=痛风 AND 提名或关键词=非布司他 OR 别嘌醇 |
