# Supplementary material for: Cardiovascular Safety of Febuxostat and Allopurinol in Hyperuricemic Patients With or Without Gout: A Network Meta-Analysis
Source: Front Med (Lausanne). 2021 Jun 15;8:698437. doi: 10.3389/fmed.2021.698437 (PMC8239361; doi:10.3389/fmed.2021.698437)
Supplement: Supplementary file 3 [file Table_3.docx]

**Table S3. Full network meta-analysis results and GRADE**

| Com | Direct evidence | | Indirect evidence | | Network meta-analysis | |
| --- | --- | --- | --- | --- | --- | --- |
|  | Odds ratio (95%CI) | Quality of evidence | Odds ratio (95%CI) | Quality of evidence | Odds ratio (95%CI) | Quality of evidence |
| **MACE** | | | | | | |
| F vs A | 0.98 (0.64, 1.5) | Moderate‡ | 0.76 (0.18, 3.4) | Low‡¶ | 0.97 (0.67, 1.37) | Moderate |
| Pl vs A | 1.5 (0.43, 5.5) | Moderate‡ | 1.9 (0.91, 4.2) | Low‡¶ | 1.79 (0.88, 3.7) | Moderate |
| Pl vs F | 2.0 (0.96, 4.1) | Moderate‡ | 1.5 (0.43, 5.7) | Low‡¶ | 1.84 (0.95, 3.67) | Moderate |
| **Non-fatal MI** | | | | | | |
| F vs A | 0.89 (0.42, 1.45) | Moderate‡ | NA | NA | 0.89 (0.42, 1.45) | Moderate |
| Pl vs A | NA | NA | 1.58 (0.41, 6.54) | Very low‡¶†† | 1.58 (0.41, 6.54) | Very low |
| Pl vs F | 1.82 (0.56, 7.05) | Moderate‡ | NA | NA | 1.82 (0.56, 7.05) | Moderate |
| **Non-fatal stroke** | | | | | | |
| F vs A | 0.97 (0.59, 1.42) | Moderate‡ | NA | NA | 0.97 (0.59, 1.42) | Moderate |
| Pl vs A | NA | NA | 1.88 (0.76, 4.64) | Very low‡¶†† | 1.88 (0.76, 4.64) | Very low |
| Pl vs F | 1.95 (0.9, 4.47) | Moderate‡ | NA | NA | 1.95 (0.9, 4.47) | Moderate |
| **Cardiovascular death** | | | | | | |
| F vs A | 1.1 (0.39, 2.3) | Moderate‡ | 0.71 (0.049, 9.9) | Low‡¶ | 1.07 (0.46, 1.93) | Moderate |
| Pl vs A | 1.5 (0.22, 11.0) | Moderate‡ | 2.2 (0.29, 17.0) | Low‡¶ | 1.75 (0.51, 6.17) | Moderate |
| Pl vs F | 1.4 (0.19, 13.0) | Moderate‡ | 1.4 (0.19, 13.0) | Low‡¶ | 1.67 (0.53, 6.14) | Moderate |
| *Limitations (risk of bias). †Inconsistency. ‡Imprecision. ¶Contributing direct evidence of moderate quality. **Contributing direct evidence of low or very low quality. ††Cannot be estimated because the drug was not connected in a loop in the evidence network. NA: not available  Each number is an odds ratio, and 95% confidence interval. A: allopurinol, F: febuxostat, Pl: placebo.  Com: Comparisons. CI: Confidence interval.  MACE: Major adverse cardiovascular events, a composite endpoint of non-fatal MI, stroke and cardiovascular death; MI: Myocardial infarction. | | | | | | |
