# Supplementary material for: Cardiovascular Safety of Febuxostat and Allopurinol in Hyperuricemic Patients With or Without Gout: A Network Meta-Analysis
Source: Front Med (Lausanne). 2021 Jun 15;8:698437. doi: 10.3389/fmed.2021.698437 (PMC8239361; doi:10.3389/fmed.2021.698437)
Supplement: Supplementary file 4 [file Table_4.docx]

**Table S4. SUCRA of each intervention in all outcomes**

| Intervention | SUCRA | | | |
| --- | --- | --- | --- | --- |
|  | MACE | non-fatal MI | non-fatal stroke | cardiovascular death |
| Allopurinol | 68.03 | 50.11 | 66.20 | 72.48 |
| Febuxostat | 77.65 | 80.72 | 77.82 | 60.31 |
| Placebo | 4.31 | 19.17 | 5.98 | 1.72 |

SUCRA: Surface under the cumulative ranking. MACE: major adverse cardiovascular events; MI: myocardial infarction.
