# Supplementary material for: Cardiovascular Safety of Febuxostat and Allopurinol in Hyperuricemic Patients With or Without Gout: A Network Meta-Analysis
Source: Front Med (Lausanne). 2021 Jun 15;8:698437. doi: 10.3389/fmed.2021.698437 (PMC8239361; doi:10.3389/fmed.2021.698437)
Supplement: Supplementary file 5 [file Table_5.docx]

**Table S5. Results of sensitivity analyses**

| Comparisons | Outcomes | | | |
| --- | --- | --- | --- | --- |
|  | MACE | Non-fatal MI | Non-fatal stroke | Cardiovascular death |
| F vs A | 1.1 (0.4, 2.8) | 0.86 (0.16, 3.6) | 0.99 (0.21, 3.2) | 1.1 (0.16, 5.1) |
| Pl vs A | 1.8 (0.56, 6.0) | 1.4 (0.14, 13.0) | 1.8 (0.23, 11.0) | 1.5 (0.16, 12.0) |
| Pl vs F | 1.7 (0.62, 4.9) | 1.6 (0.33, 9.3) | 1.8 (0.50, 7.3) | 1.4 (0.18, 13.0) |

Each number is an odds ratio (95% confidence interval).
