# Supplementary material for: Cardiovascular Safety of Febuxostat and Allopurinol in Hyperuricemic Patients With or Without Gout: A Network Meta-Analysis
Source: Front Med (Lausanne). 2021 Jun 15;8:698437. doi: 10.3389/fmed.2021.698437 (PMC8239361; doi:10.3389/fmed.2021.698437)
Supplement: Supplementary file 7 [file Data_Sheet_1.docx]

**R code for this network meta-analysis**

#network plot

library(multinma)

options(mc.cores = parallel::detectCores())

library(readxl)

outcome <- read_excel("filename")

net <- set_agd_arm(outcome, study = study, trt = trt, r = r, n = n, trt_class = trt_class)

plot(net, weight_nodes = TRUE, weight_edges = TRUE, show_trt_class = TRUE) +

ggplot2::theme(legend.position = “bottom”, legend.box = “vertical”)

#network meta-analysis

library("gemtc")

library("rjags")

data <- read.csv(“filename", sep=",", header=T)
treatments <- read.csv(“filename", sep=",", header=T)

network <- mtc.network(data, description="Example", treatments=treatments)

model <-mtc.model(network, type = "consistency", factor = 2.5, n.chain = 4,linearModel="random")

results <- mtc.run(model, sampler = NA, n.adapt = 40000, n.iter = 160000, thin = 1)

#forest plot

forest(relative.effect(results, “name of treatment”))

#league table

mtcresults = as.data.frame(round(exp(relative.effect.table(results)),2))

write.csv(mtcresults, file="{file location}")

#probability of rank

rank.prob<- rank.probability(results, preferredDirection=-1)

summary(rank.prob)

#SUCRA

cumrank.prob <- apply(t(rank.prob), 2, cumsum)

sucra <- round(colMeans(cumrank.prob[-nrow(cumrank.prob),]),4)

write.csv(sucra, file="{file location}")

#nodesplit analysis

result<-mtc.nodesplit(network,likelihood="binom",link="logit",linearModel="random",n.chain =4,n.adapt=40000, n.iter=160000, thin=1)

plot(summary(result))

#heterogeneity

result.anohe<- mtc.anohe(network, n.chain=4, n.adapt=40000, n.iter=160000, thin=1)

plot(summary(result.anohe))
