# Supplementary material for: Cardiovascular Safety of Febuxostat and Allopurinol in Hyperuricemic Patients With or Without Gout: A Network Meta-Analysis
Source: Front Med (Lausanne). 2021 Jun 15;8:698437. doi: 10.3389/fmed.2021.698437 (PMC8239361; doi:10.3389/fmed.2021.698437)
Supplement: Supplementary file 8 [file Data_Sheet_2.docx]

**Figure legends for supplementary materials**

**Figure S1. Risk of bias summary**

Symbol
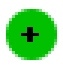
 represents low risk, symbol
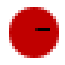
 represents high risk, and symbol
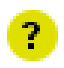
 represents unclear risk.

**Figure S2. Forest plots of node split analysis for different outcomes**

P-value > 0.05 indicates that consistency between direct analysis and indirect analysis is good. A: MACE (major adverse cardiovascular events); B: cardiovascular death.

**Figure S3. Forest plots of heterogeneity for different outcomes**

I^2 < 50% indicates heterogeneity is low. A: MACE (major adverse cardiovascular events); B: non-fatal MI (myocardial infarction), C: non-fatal stroke, D: cardiovascular death.
