# Supplementary figures and images for: Cardiovascular Safety of Febuxostat and Allopurinol in Hyperuricemic Patients With or Without Gout: A Network Meta-Analysis
Source: Front Med (Lausanne). 2021 Jun 15;8:698437. doi: 10.3389/fmed.2021.698437 (PMC8239361; doi:10.3389/fmed.2021.698437)

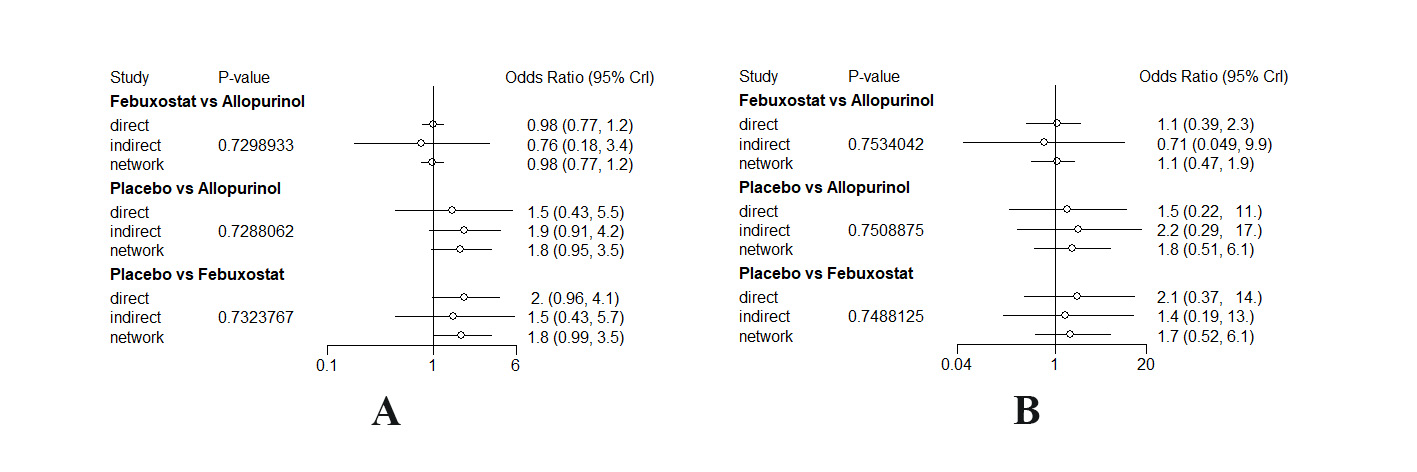

Supplement: Supplementary file 10 [file Image_2.JPEG]

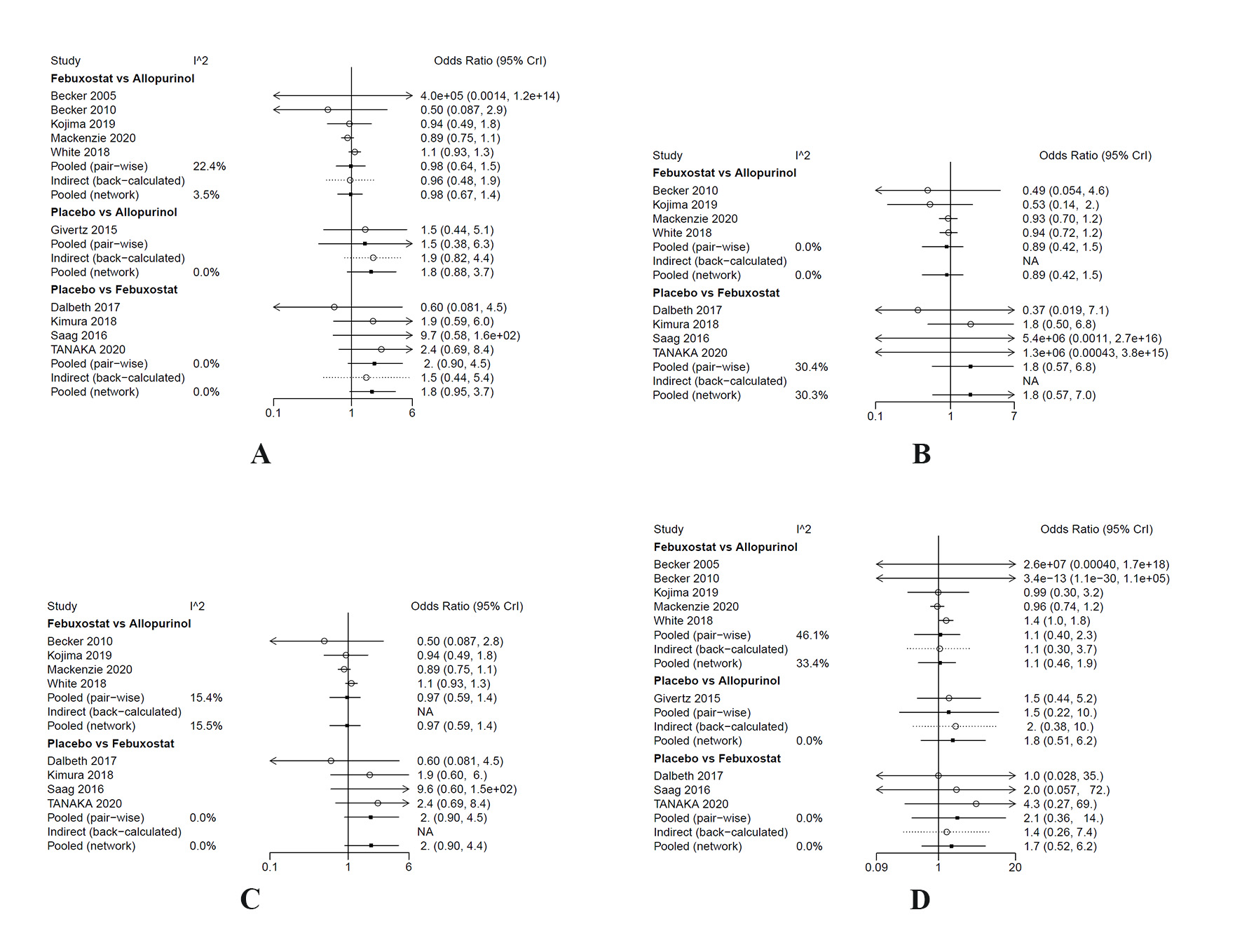

Supplement: Supplementary file 11 [file Image_3.JPEG]
